# The impact of multipollutant exposure on hepatic steatosis: a machine learning-based investigation into multipollutant synergistic effects

**DOI:** 10.3389/fpubh.2025.1598639

**Published:** 2025-05-22

**Authors:** Chunying Yan, Zhanfang Zhu, Xueyan Guo, Wei Zong, Guisheng Liu, Yan Jin, Shiyuan Cui, Fuqiang Liu, Shujuan Gao

**Affiliations:** ^1^Department of Gastroenterology, Shaanxi Provincial People's Hospital, Xi’an, China; ^2^Xi'an Jiaotong University Hospital, Xi’an, China; ^3^Department of Cardiology, Shaanxi Provincial People's Hospital, Xi’an, China

**Keywords:** machine learning, percentage of liver fat (PLF), heavy metals, polycyclic aromatic hydrocarbons (PAHs), volatile organic compounds (VOCs)

## Abstract

**Introduction:**

This study examines the synergistic effects of multi-pollutant exposure on hepatic lipid accumulation in non-alcoholic fatty liver disease (NAFLD) through the application of an explainable machine learning framework. This approach addresses the limitations of traditional models in managing complex environmental interactions.

**Methods:**

Using data from the National Health and Nutrition Examination Survey (NHANES) 2015–2016 (n = 494), we developed a stacked ensemble model that integrates LASSO, support vector machines (SVM), neural networks, and XGBoost to analyze urinary biomarkers of heavy metals, polycyclic aromatic hydrocarbons (PAHs), and volatile organic compounds (VOCs). The Environmental Pollution Exposure Index (EPEI) was constructed to quantify cumulative effects, with SHAP values employed to identify critical pollutants and thresholds. Subgroup analyses were conducted to assess heterogeneity across different Body Mass Index (BMI), diabetes, and hyperlipidemia statuses.

**Results:**

2-Hydroxynaphthalene was identified as the predominant pollutant (SHAP = 0.89), with cobalt and VOC metabolites (e.g., N-Acetyl-S-(2-carbamoylethyl)-L-cysteine) also contributing significantly. The EPEI demonstrated strong associations with obesity-related parameters (PLF: 7.02 vs. 3.41 in high/low-exposure groups, *p* < 0.0001) and hyperlipidemia (OR = 2.28 vs. 1.08, *p* = 2.7e-06). The model demonstrated an amplification of effects in subgroups with severe obesity (OR = 2.66, 95% CI: 2.08–3.24) and impaired fasting glucose.

**Discussion:**

This study establishes a machine learning framework for assessing multi-pollutant risks in NAFLD, identifying 2-Hydroxynaphthalene as a significant hepatotoxicant and EPEI as a quantifiable metric of exposure. The findings highlight the metabolic vulnerabilities associated with obesity and early dysglycemia, thereby informing precision prevention strategies. Methodological advancements integrate exposomics with interpretable artificial intelligence, facilitating targeted interventions in environmental health.

## 1 Introduction

Non-alcoholic fatty liver disease (NAFLD), a predominant contributor to the global burden of chronic liver diseases, has surpassed the conventional understanding centered on metabolic risk factors in its pathogenesis. Increasing attention is being paid to the pathogenic influence of environmental pollutant exposure (1–3). Epidemiological research indicates that urinary metabolites of heavy metals, polycyclic aromatic hydrocarbons (PAHs), and volatile organic compounds (VOCs) may perturb hepatic lipid metabolic homeostasis through various mechanisms (4).

Mitochondrial dysfunction is a crucial pathway linked to insulin resistance and metabolic syndrome, triggered by persistent organic pollutants (POPs) like PCBs and PAHs. Low doses of bisphenol A (BPA) also cause lipid buildup in liver cells due to ROS from mitochondrial damage. Additionally, PAHs induce lipid peroxidation, leading to oxidative stress and immune system changes. Urinary metabolites of PAHs, such as benzo[a]pyrene, are associated with increased lipid peroxidation products, potentially worsening liver cell membrane damage and inflammation (5–8). Inflammatory pathways further underscore the influence of PAHs and VOCs on hepatic lipid metabolism. Exposure to PAHs is linked to elevated levels of inflammatory markers, such as interleukin (IL)-6 and IL-8, which may impair liver function by enhancing oxidative stress and inflammatory pathways (9). Similarly, urinary metabolites of VOCs are associated with biomarkers of oxidative stress, indicating that VOCs may adversely affect liver health through the induction of systemic oxidative stress (10). Various studies have examined the combined impact of PAHs and metals on oxidative stress. Research in Caofeidian, China, found that co-exposure to these substances was positively linked to oxidative stress markers like 8-OHdG and 8-iso-PGF2α, suggesting a potential contribution to liver dysfunction. Additionally, VOCs have been studied for their role in liver issues, such as NAFLD (11). A study on Korean adolescents showed that low-level exposure to VOCs and PAHs was associated with higher ALT activity and NAFLD prevalence, indicating that these compounds may harm liver health through oxidative stress pathways (12).

.Traditional generalized linear models, such as logistic regression, are inadequate for addressing the nonlinear interactions and multicollinearity present in high-dimensional data, thus limiting their effectiveness in analyzing the joint effects of multiple pollutants. This study introduces an innovative approach by employing a stacked ensemble learning framework (13–15). Initially, base models—including LASSO regression (with *λ* optimized via 10-fold cross-validation) (16), support vector machine (SVM) with a radial basis function kernel (17), and a neural network (NN) with a single hidden layer (18)—are employed to capture localized nonlinear relationships and sparse associations between pollutants and percentage of liver fat (PLF) (19). Subsequently, XGBoost (20) is employed as the meta-model to synthesize predictions from base models, utilizing its gradient-boosted tree algorithm to unravel complex interactions among pollutants. To enhance interpretability, Shapley Additive exPlanations (SHAP) values are employed to quantify the marginal contributions of individual pollutants and to identify critical risk substances with specific exposure thresholds (21).

Nevertheless, existing research predominantly emphasizes single-pollutant analyses, leaving the synergistic effects of combined exposure to heavy metals, PAHs, and VOCs in real-world environments largely unexplored. This gap significantly impedes the advancement of environmental risk models for NAFLD. Drawing on NHANES 2015–2016 data, this study pioneers the systematic integration of urinary heavy metals, PAHs, and VOCs metabolites to assess their combined effects on PLF through the proposed machine learning framework. This methodological innovation not only addresses the limitations of traditional statistical models but also lays a theoretical foundation for developing exposomics-based precision prevention strategies for NAFLD.

## 2 Methods

### 2.1 Data provenance and processing

The National Health and Nutrition Examination Survey (NHANES)^1^ is a nationally representative cross-sectional surveillance program administered by the National Center for Health Statistics (NCHS) under the auspices of the Centers for Disease Control and Prevention (CDC). Initiated in the 1960s, this program systematically assesses the health and nutritional status of both adult and pediatric populations in the United States. It employs a multi-stage stratified random sampling methodology to ensure that the samples accurately reflect the diverse geographic regions, racial groups, and age cohorts within the U.S. population. In the present study, data from the 2015–2016 survey cycle were utilized. The National Center for Health Statistics (NCHS) research ethics review board (ERB) approved the NHANES study protocol, and participants provided written informed consent at enrollment. The NCHS Institutional Review Board/ethics review board (IRB/ERB) protocol numbers of 2015–2016 National Health and Nutrition Survey is “#2011–17.” The inclusion criteria required participants to have complete datasets concerning urinary heavy metals, VOCs, PAHs, and urinary creatinine measurements. To reduce confounding variables and enhance the validity of the analysis, individuals lacking data on diabetes mellitus or dyslipidemia diagnoses were excluded. Following these selection procedures, a final cohort of 494 participants was included in the statistical analysis. PLF was calculated according to methodologies detailed in a previously published study (19). For basic clinical information, we used multiple imputation to fill in missing values. Due to the fact that creatinine is not reabsorbed after glomerular filtration, it can be used as a quantitative biomarker for detecting glomerular filtration rate or renal excretion function. In practical operation, it is sometimes difficult to collect urine samples for a long time, and there are many influencing factors during storage. Therefore, the ratio of biomarkers to creatinine in random urine samples can be used as a detection indicator, which can more conveniently and effectively reflect the true situation of patients and facilitate sampling. Concentrations of urinary heavy metals, VOCs, and PAHs were normalized to urinary creatinine levels to account for variations in urine dilution, with final values expressed in micrograms per gram of creatinine (μg/g creatinine).

### 2.2 Machine learning and visualization

In this study, we employed a machine learning-based ensemble learning framework to analyze the complex relationship between environmental exposures and PLF. Initially, we extracted heavy metals, VOCs, and PAHs adjusted for creatinine as features from the filtered dataset, with continuous PLF serving as the target variable. To develop robust predictive models, we trained three base learners—SVM, LASSO regression, and a neural network—and assessed their performance using five-fold cross-validation. Subsequently, we constructed a stacked ensemble model that takes the predicted values of the base learners as input features, utilizes XGBoost as a meta model to integrate multi-source information, and based on this, constructs the Environmental Pollution Exposure Index (EPEI). Our study implements a nested cross-validation framework (5 × 5-fold) for model optimization: the inner loop conducts hyperparameter selection via randomized search, while the outer loop evaluates generalization performance. Specifically, the support vector machine (SVM) optimizes radial basis function kernel parameters through a customized grid search (*σ*: 10^−3^–10^1^, C: 10^−2^–10^2^). LASSO regression fixes *α* = 1 and tunes regularization strength *λ* across an extended logarithmic range (10^−5^–10^2^). The neural network systematically adjusts hidden layer neurons (3–15 with incremental steps of 3) and L2 weight decay coefficients (10^−4^–10^0^). For meta-modeling, XGBoost utilizes base model predictions as explanatory variables and automatically optimizes tree parameters via outer cross-validation (tuneLength = 5). All base models undergo standardization preprocessing. Both nested layers employ a five-fold stratified cross-validation design to ensure independence between parameter selection and performance evaluation phases, thereby preventing information leakage. To interpret the model and evaluate feature importance, we employed SHAP values. Derived from SHAP values in cooperative game theory, SHAP values quantify the contribution of each feature to the predictions. We calculated SHAP values for both the meta-model and the base learners to identify the top 10 features critical for predictions, and subsequently visualized these findings.

$\mathrm{Let}\;\mathrm{the base model}\;\mathrm{set}\;\mathrm{be}\;\varkappa =\left\{h_{\mathrm{svn}}{\mathrm{,h}}_{{\mathrm{LA}}_{\mathrm{SS}}O}{\mathrm{,h}}_{\mathrm{NN}}\right\}$ , whose predictions form the feature vector:

$$z\left(x\right)={\left[h_{SVM}\left(x\right),h_{L}ASSO^{\left(x\right)},h_{NN}\left(x\right)\right]}^{T}\in R^{3}$$

The meta-model learns a nonlinear mapping via XGBoost:

$f:R^{3}\to R$ , where f(·)= XGBoost model


ThefinalEPEIisdefinedas:EPEI(x)=f(z(x))

For the purpose of visualization, we employed beeswarm and pie charts. The beeswarm plot effectively demonstrates the distribution of SHAP values across various features, thereby elucidating the relationship between feature values and model outputs. Meanwhile, the pie chart provides an intuitive representation of the importance ranking of features. These visualizations facilitate our understanding of the impact of environmental exposures on PLF, thereby providing a foundation for further analyses.

### 2.3 Subgroup analysis strategy

This study utilized a stratified analysis approach to investigate the heterogeneity in the relationship between EPEI and PLF across various population subgroups. Utilizing data from the NHANES, we incorporated gender, Body Mass Index (BMI) categories, diabetes status, and hyperlipidemia status as stratification variables to develop a weighted survey design object (weight variable: WTFSM, cluster variable: sdmvpsu, strata variable: sdmvstra). Initially, we established a baseline linear regression model and conducted interaction testing using analysis of variance. Specifically, we employed a likelihood ratio test to compare the full model (including interaction terms) with the reduced model (containing only main effects) to determine interaction *p*-values. The subgroups were delineated as follows: ① gender (male/female); ② BMI categories [underweight (BMI < 18.5), normal (18.5 ≤ BMI < 25), overweight (25 ≤ BMI < 30), obese (30 ≤ BMI < 35), severely obese(BMI ≥ 35)]; ③ diabetes status [normal, impaired fasting glucose (IFG) fasting plasma glucose: 6.1–7.0 mmol/L; 2-h postprandial plasma glucose: within the normal range], impaired glucose tolerance (IGT) (Fasting blood glucose:< 7.0 mmol/L; 2-h postprandial blood glucose: 7.8 ~ 11.1 mmol/L), diagnosed diabetes (DM); and ④ hyperlipidemia status (yes/no).

### 2.4 Model evaluation and statistical workflow

To evaluate the predictive performance of the model, we employed weighted receiver operating characteristic (ROC) curve analysis to assess the discriminatory capacity of EPEI for detecting PLF abnormalities (>5). When calculating the SHAP value, we used kernelshap and SHAPforxgboost to meet different requirements. This involved calculating the area under the curve (AUC) for each subgroup and comparing inter-group differences using the Bootstrap method. All analyses were conducted using R version 4.2.3, with the primary use of the nhanesR package for diagnosing comorbidities and managing complex sampling designs, and ggplot2 for data visualization. The statistical analysis adhered to the following procedure (1): Data preprocessing, which included converting ordinal categorical variables to numerical format and standardizing continuous variables (2); Establishment of a weighted linear regression model (3); Testing for interaction significance using ANOVA (4); Estimation of stratum-specific effect sizes along with 95% confidence intervals (5); Visualization of subgroup-specific effects using forest plots with annotated interaction *p*-values. All model parameter estimates incorporated sampling weights to ensure that the results were representative.

## 3 Results

### 3.1 Machine learning and SHAP interpretation

**Figure 1:**
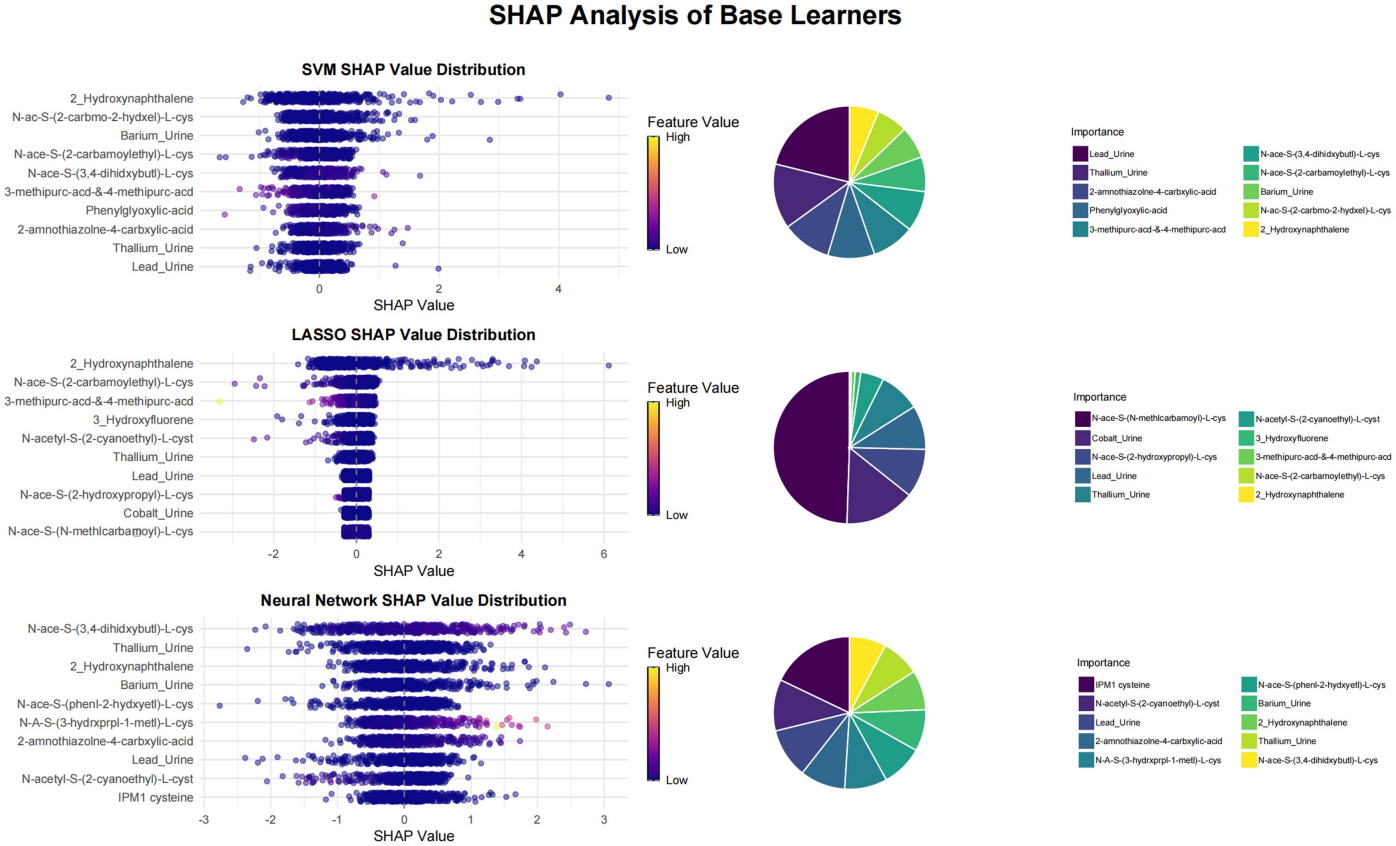
Visualization of SHAP analysis for top 10 features in meta learner. The swarm plot ranks features by their average SHAP values, with color indicating the magnitude of the feature values and the position of the points determined by the SHAP values. A pie chart is utilized to illustrate the contribution proportion of each feature to the model’s prediction results, where each sector corresponds to a feature, and its area reflects the feature’s relative importance in the model’s predictions.

**Figure 2:**
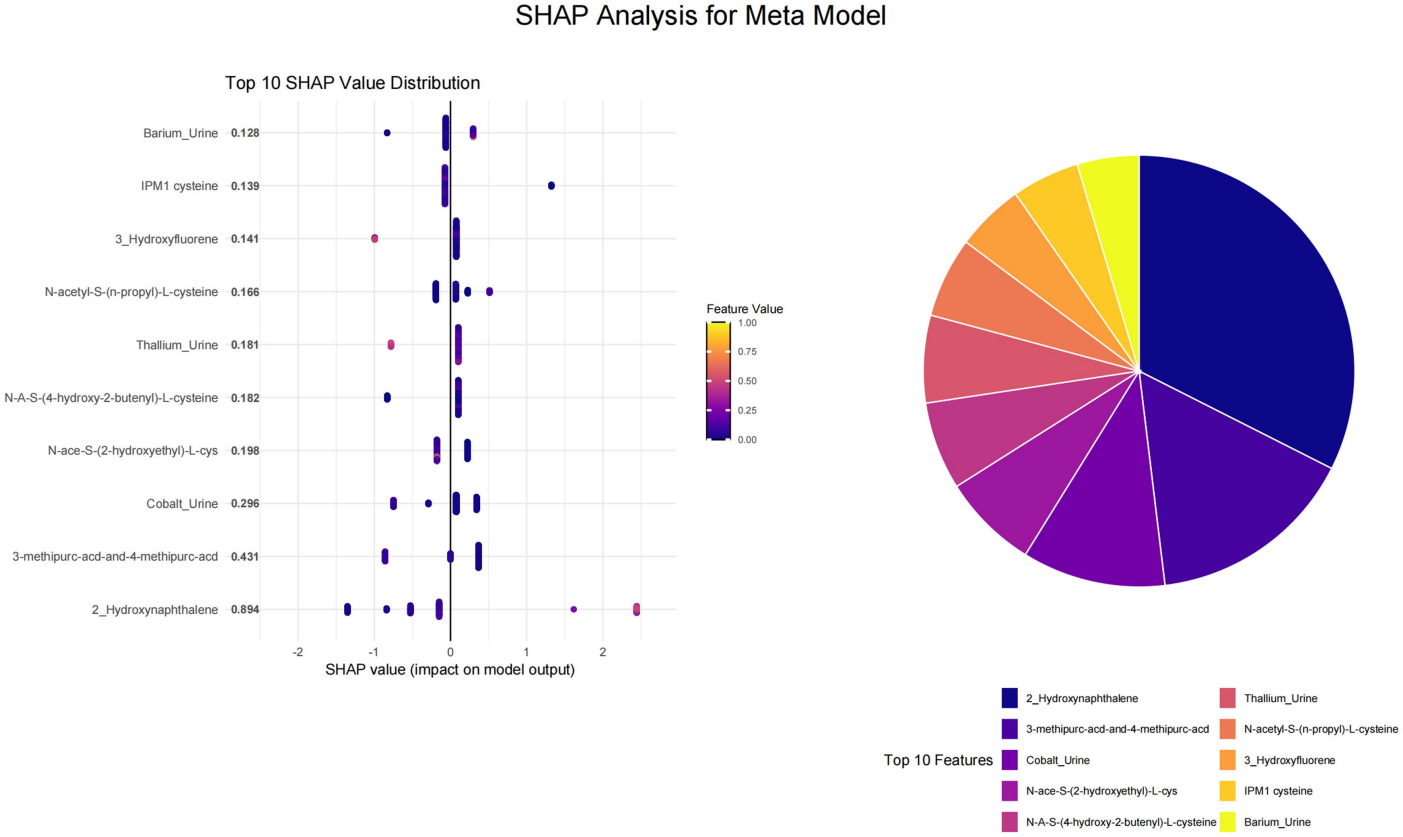
Visualization of SHAP analysis for top 10 features in each base learner.

This study utilized SHAP value analysis to elucidate the contributions of various features across different machine learning models in predicting outcomes. In the meta-model analysis, 2-Hydroxynaphthalene exhibited the highest SHAP value of 0.8939, indicating a substantial impact on model predictions (Figure 1). The features 3-methipurc-acd-&-4-methipurc-acd and Cobalt demonstrated SHAP values of 0.4306 and 0.2962, respectively, underscoring their relatively high importance in the model. Within the base learners (Figure 2), the SVM model assigned a SHAP value of 0.4278 to 2-Hydroxynaphthalene and 0.2798 to N-Acetyl-S-(2-carbamoylethyl)-L-cysteine. In the LASSO model, 2-Hydroxynaphthalene had a SHAP value of 0.6454, while N-Acetyl-S-(2-carbamoylethyl)-L-cysteine had a value of 0.1957. In the neural network model, N-Acetyl-S-(3,4-dihydroxybutyl)-L-cysteine exhibited a SHAP value of 0.6233, whereas 2-Hydroxynaphthalene had a value of 0.3789. These findings highlight variations in feature importance across different models; however, 2-Hydroxynaphthalene consistently demonstrated high SHAP values.

### 3.2 Correlation analysis

**Figure 3:**
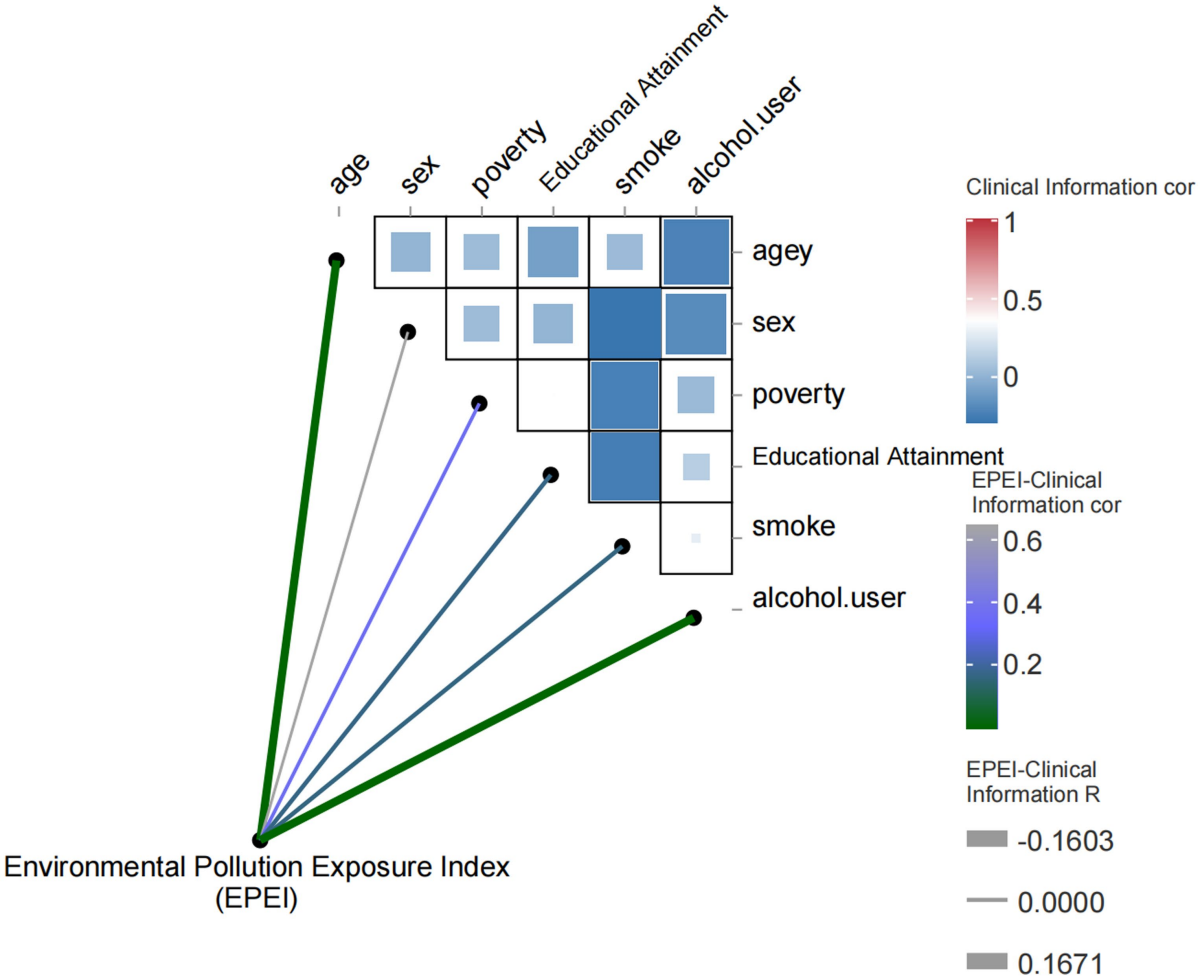
Heatmap of correlation between environmental pollution exposure index (EPEI) and clinical characteristics.

In this study, we developed EPEI using meta-modeling analysis to investigate its association with clinical variables and environmental pollutants. The study encompassed a range of clinical variables, including age, gender, poverty status, education level, smoking habits, and alcohol consumption, in addition to various environmental pollutant indicators. In the correlation analysis of clinical variables (Figure 3), the EPEI demonstrated a significant positive correlation with age (correlation coefficient = 0.17, *p*-value = 0.00019), suggesting that exposure to environmental pollution may increase with advancing age. Furthermore, the EPEI exhibited a significant negative correlation with alcohol consumption (correlation coefficient = −0.22, *p* = 0.00035), indicating a lower risk of pollution exposure among individuals with higher frequencies of alcohol use.

**Figure 4:**
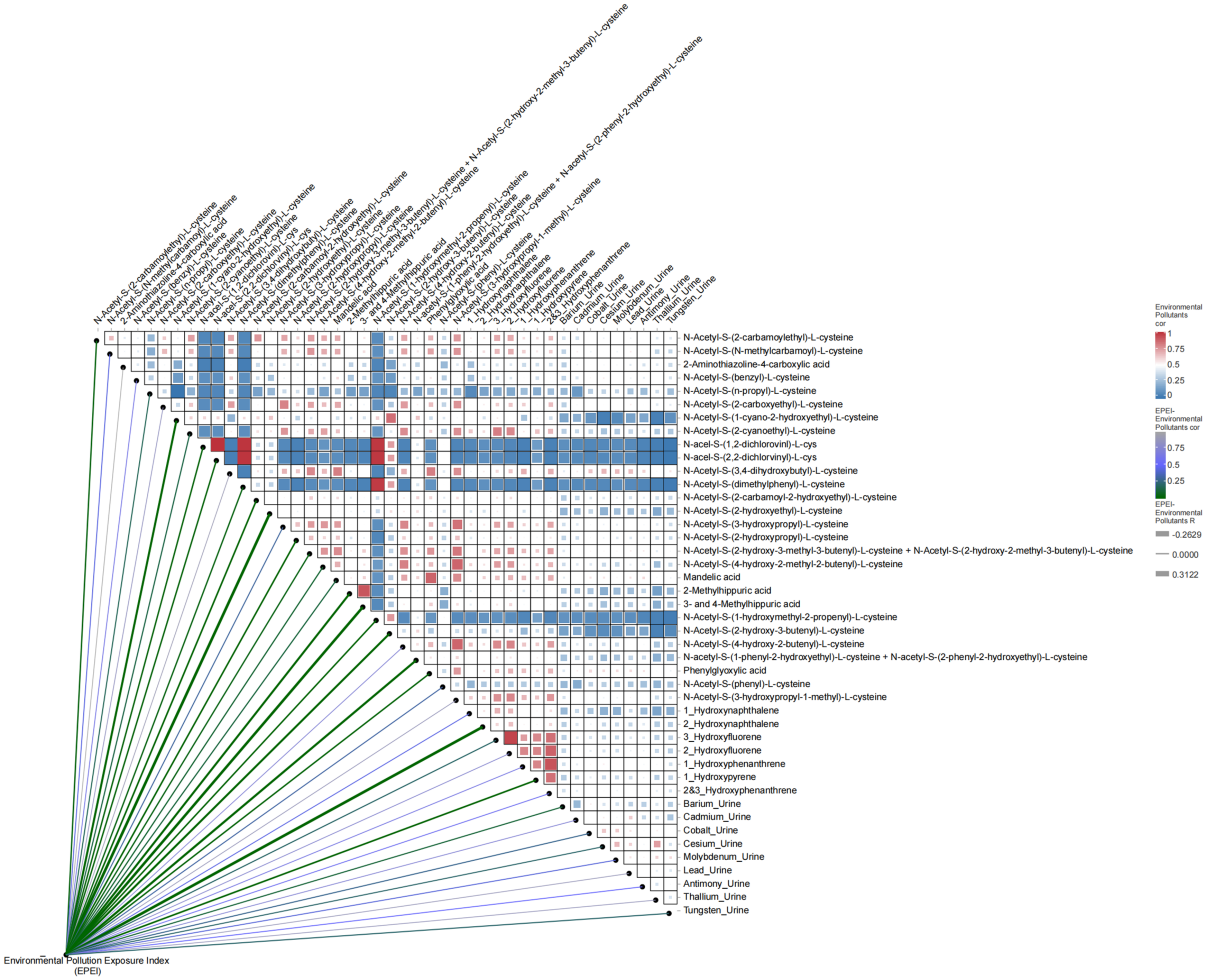
Heatmap of correlation between environmental pollution exposure index (EPEI) and environmental pollutants.

In relation to environmental pollutants (Figure 4), EPEI demonstrated significant correlations with various pollutants, including N-Acetyl-S-(2-carbamoylethyl)-L-cysteine (correlation coefficient = −0.095, *p* = 0.035) and N-Acetyl-S-(N-methylcarbamoyl)-L-cysteine, suggesting their potential role as indicators in comprehensive pollution assessment. Further analysis identified correlations among clinical variables, such as age and gender (correlation coefficient = 0.012) and poverty status and education level (correlation coefficient = 0.35), highlighting the extensive influence of socioeconomic factors on health outcomes. Additionally, environmental pollutants exhibited intricate correlation networks, exemplified by the relationships between N-Acetyl-S-(2-carbamoylethyl)-L-cysteine and N-Acetyl-S-(N-methylcarbamoyl)-L-cysteine (correlation coefficient = 0.65), as well as N-Acetyl-S-(benzyl)-L-cysteine and N-Acetyl-S-(n-propyl)-L-cysteine (correlation coefficient = 0.42), which may be attributed to shared exposure sources or analogous metabolic pathways.

### 3.3 Demographic and clinical characteristics of the study population

This study included 494 participants for whom comprehensive data on environmental pollutant exposure and metabolic disorder diagnoses were available. EPEI was developed using machine learning techniques, enabling the classification of participants into high- and low-exposure groups (Table 1). Baseline analysis indicated that the mean age of the entire cohort was 47.44 years (95% CI: 45.44–49.45), with no statistically significant age difference observed between the exposure groups (*p* = 0.53). Notable differences were identified in obesity-related parameters, with the high-exposure group exhibiting significantly higher values compared to the low-exposure group in terms of BMI (31.78 vs. 27.40 kg/m^2^), PLF (7.02 vs. 3.41), and EPEI (6.42 vs. 4.18), all with *p*-values less than 0.0001. Analysis of categorical variables revealed a higher prevalence of hyperlipidemia in the high-exposure group (51.16% vs. 28.02%, *p* = 0.01). Furthermore, the classification of severe obesity was associated with a substantially increased exposure risk (70.80% vs. 10.54%, *p* < 0.0001). The distribution of educational attainment revealed non-significant trends, with 52.64% of individuals in the high-exposure group having an education level of high school or less, compared to 63.57% of those with some college education in the low-exposure group (*p* = 0.14). No significant differences were observed between the groups in terms of gender distribution (*p* = 0.77), smoking status (*p* = 0.88), or alcohol consumption (*p* = 0.16). Although 56.32% of confirmed DM cases were found in the high-exposure group, this trend did not reach statistical significance (*p* = 0.07). Collectively, these findings suggest strong associations between elevated exposure to environmental pollutants and obesity-related metabolic disturbances, particularly hyperlipidemia, while indicating weaker correlations with demographic and behavioral factors. This pattern implies potential mechanistic pathways through which exposure to environmental pollutants may impact health outcomes via metabolic dysregulation.

**Table 1:** Baseline characteristics of participants stratified by the median score of environmental pollution exposure index (EPEI).

Characteristics	Total	Environmental pollution exposure index		*P*-value
		Down	High	
Age [mean(CI)]	47.44(45.44, 49.45)	46.79(44.12, 49.46)	48.26(44.98, 51.53)	0.53
Poverty [mean(CI)]	2.99(2.67, 3.31)	3.14(2.77, 3.52)	2.79(2.35, 3.23)	0.19
EPEI [mean(CI)]	5.18(4.93, 5.43)	4.18(4.09, 4.27)	6.42(6.25, 6.59)	< 0.0001
PLF [mean(CI)]	5.02(4.58, 5.46)	3.41(2.97, 3.85)	7.02(6.20, 7.84)	< 0.0001
BMI_kg.m^2^ [mean(CI)]	29.34(28.51, 30.18)	27.40(26.54, 28.25)	31.78(30.91, 32.66)	< 0.0001
Edu [%(SE)]				0.14
No more than 12th (includes 12th grade with no diploma)	14.70(0.02)	47.36(8.23)	52.64(8.23)	
High school grad/GED or equivalent	23.05(0.04)	44.25(7.57)	55.75(7.57)	
Some college or AA degree (includes more than high school)	27.02(0.04)	63.57(6.31)	36.43(6.31)	
College graduate or above	35.22(0.09)	59.75(6.03)	40.25(6.03)	
Sex [%(SE)]				0.77
Male	50.32(0.07)	54.62(5.40)	45.38(5.40)	
Female	49.68(0.09)	56.16(4.48)	43.84(4.48)	
Smoke [%(SE)]				0.88
Never	57.42(0.09)	56.86(4.43)	43.14(4.43)	
Former	22.30(0.05)	53.77(11.52)	46.23(11.52)	
Now	20.28(0.03)	52.97(6.07)	47.03(6.07)	
Alcohol.user [%(SE)]				0.16
Never	10.77(0.02)	57.14(8.96)	42.86(8.96)	
Former	12.72(0.03)	36.25(4.40)	63.75(4.40)	
Mild	41.05(0.08)	57.94(5.88)	42.06(5.88)	
Moderate	15.29(0.04)	62.64(8.57)	37.36(8.57)	
Heavy	20.17(0.03)	55.81(7.03)	44.19(7.03)	
Hyperlipidemia [%(SE)]				0.01
No	28.28(0.05)	71.98(5.05)	28.02(5.05)	
Yes	71.72(0.10)	48.84(4.94)	51.16(4.94)	
DM [%(SE)]				0.07
DM	19.16(0.03)	43.68(7.32)	56.32(7.32)	
IFG	14.19(0.04)	45.51(8.94)	54.49(8.94)	
IGT	4.95(0.02)	63.19(10.20)	36.81(10.20)	
No	61.70(0.10)	60.67(4.16)	39.33(4.16)	
BMI_category [%(SE)]				< 0.0001
Underweight	1.38(0.01)	89.46(10.27)	10.54(10.27)	
Normal	26.39(0.05)	70.32(5.36)	29.68(5.36)	
Overweight	35.51(0.06)	62.37(5.52)	37.63(5.52)	
Obese	17.28(0.03)	45.35(8.15)	54.65(8.15)	
Severely obese	18.92(0.04)	29.20(5.79)	70.80(5.79)	

EPEI, environmental pollution exposure index; PLF, liver fat percentage; IFG, impaired fasting glucose; IGT, impaired glucose tolerance; DM, diagnosed diabetes; BMI, Body Mass Index.

### 3.4 Receiver operating characteristic curve

**Figure 5:**
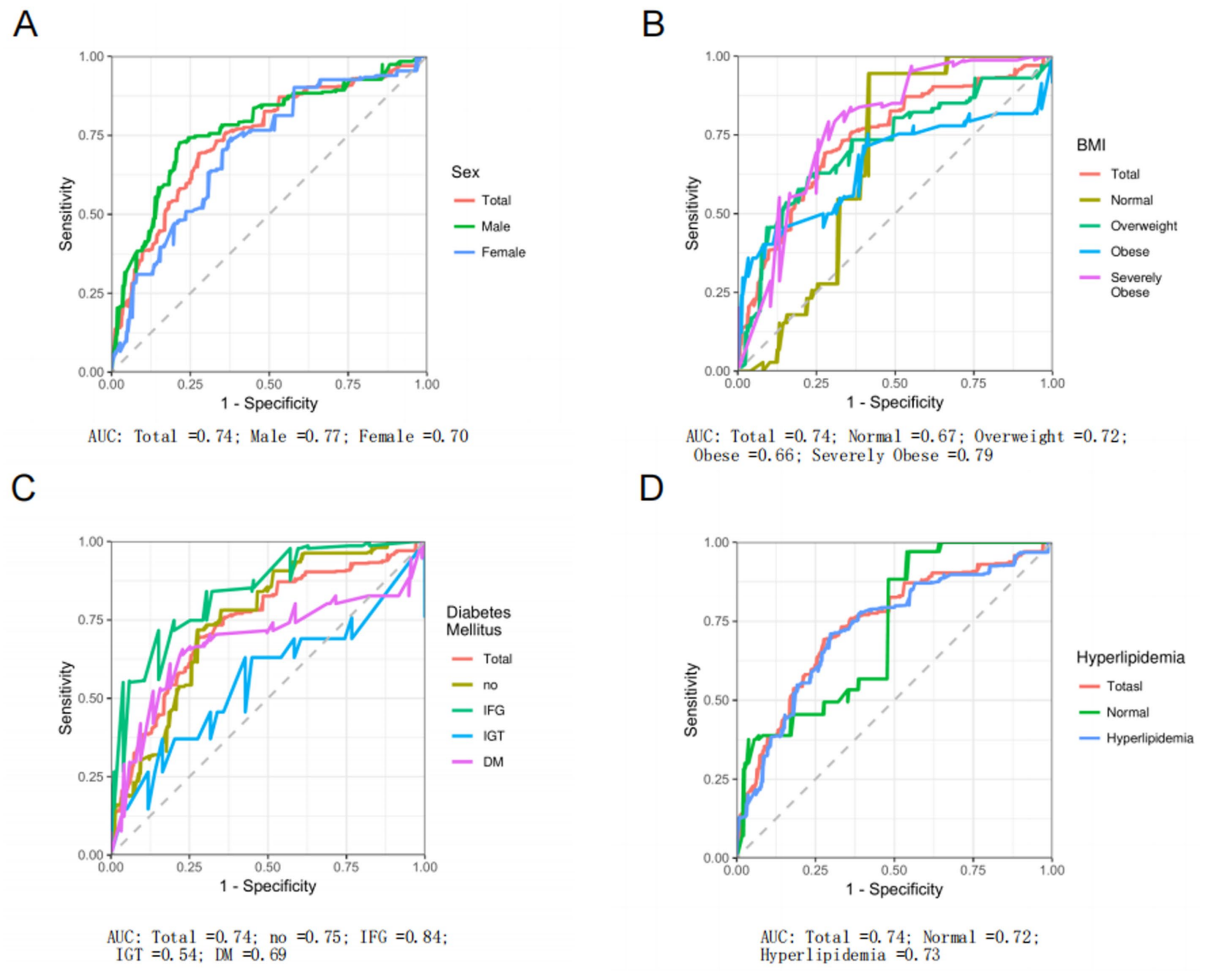
Receiver operating characteristic (ROC) curve analysis of environmental pollution exposure index (EPEI) for liver fat percentage (PLF). This analysis evaluates the diagnostic performance of EPEI for PLF across different subgroups. Specifically, it includes comparisons between: **(A)** the total population and different gender subgroups; **(B)** the total population and subgroups categorized by different BMI classifications; **(C)** the total population and subgroups with different glucose metabolism levels; and **(D)** the total population and subgroups with and without hyperlipidemia.

The analysis of the ROC for EPEI in diagnosing PLF across various subgroups yields heterogeneous results. Overall, the EPEI exhibits moderate diagnostic efficacy for PLF across diverse demographic and clinical subgroups, with Area Under the Curve (AUC) values predominantly ranging from acceptable to good. Notably, a discernible variation in diagnostic performance is observed between sexes. The male subgroup demonstrates a slightly higher AUC (AUC = 0.77) compared to the female subgroup (AUC = 0.70) (Figure 5A), suggesting that the EPEI may possess enhanced discriminatory power for PLF in males. This disparity may be attributed to biological or physiological differences between sexes that affect the impact of pollution exposure on liver fat accumulation. In relation to BMI categories, the diagnostic efficacy of EPEI demonstrates variability. Notably, the severely obese subgroup exhibits the highest AUC (AUC = 0.79) among all BMI subgroups (Figure 5B), indicating that EPEI is particularly proficient in differentiating PLF levels within this population. Conversely, the obese subgroup presents a comparatively lower AUC (AUC = 0.66), which may suggest that additional factors, such as obesity-related comorbidities, could be confounding the association between pollution exposure and liver fat in this group. Concerning DM subgroups (Figure 5C), the performance of EPEI varies significantly. The IFG subgroup demonstrates the highest AUC (AUC = 0.84), signifying that EPEI is an effective diagnostic tool for PLF at this early stage of glucose metabolism disturbance. In contrast, the IGT subgroup exhibits a substantially lower AUC (AUC = 0.54), potentially reflecting the intricate metabolic alterations in this group that may obscure the impact of pollution exposure on liver fat. The normal and DM subgroups are intermediate, with the DM subgroup displaying a lower AUC (AUC = 0.69) than the normal subgroup (AUC = 0.75), suggesting that the diagnostic utility of EPEI diminishes as diabetes progresses. Lastly, in the context of hyperlipidemia (Figure 5D), EPEI performs similarly in both the presence (AUC = 0.73) and absence (AUC = 0.72) of the condition, with only a marginal difference in AUC values. This implies that lipid abnormalities may not significantly modulate the relationship between pollution exposure and PLF.

### 3.5 Subgroup analysis

**Figure 6:**
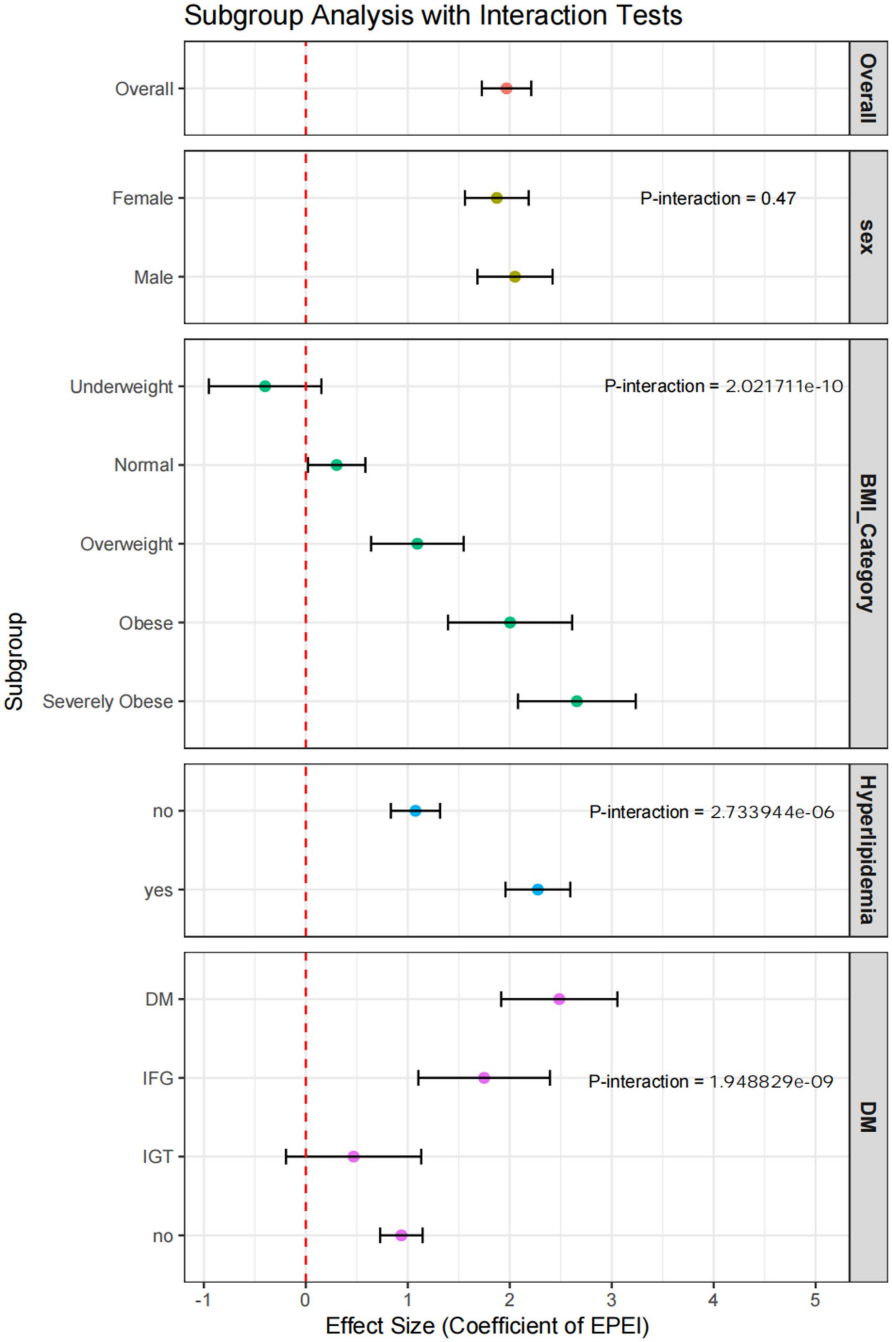
Forest plot of subgroup analysis incorporating interaction tests. This figure presents the results of subgroup analyses adjusted for interaction tests, visually representing the effect sizes and confidence intervals for each subgroup.

Subgroup analyses indicated notable variations in the association between EPEI and PLF across different demographic and clinical categories (Figure 6). In terms of sex, EPEI significantly influenced PLF in both males (OR = 2.05, 95% CI: 1.68–2.42) and females (OR = 1.87, 95% CI: 1.56–2.19); however, the interaction *p*-value is 0.47, suggesting that sex did not exert a statistically significant moderating effect on the EPEI-PLF relationship. Regarding BMI categories, the effect of EPEI on PLF intensified with increasing BMI, particularly within the severely obese subgroup (OR = 2.66, 95% CI: 2.08–3.24), with an interaction p-value of 2.0e-10, indicating a significant modifying effect of BMI. Concerning diabetes status, EPEI had a significant impact on PLF in both the DM subgroup (OR = 2.49, 95% CI: 1.92–3.06) and the IFG subgroup (OR = 1.75, 95% CI: 1.10–2.39), but not in the IGT subgroup (OR = 0.47, 95% CI: −0.19-1.13). The interaction p-value of 1.9e-09 further underscores the significant modifying effect of diabetes status on this relationship. Within the subgroup characterized by hyperlipidemia status, the effect of EPEI on PLF exhibited a statistically significant difference between individuals with hyperlipidemia (OR = 2.28, 95% CI: 1.96–2.59) and those without the condition (OR = 1.08, 95% CI: 0.83–1.32). The interaction *p*-value of 2.7e-06 indicates a significant modifying effect attributable to hyperlipidemia status.

## 4 Discussion

This study conducted a systematic investigation into the synergistic effects of multi-pollutant exposure on PLF using an advanced machine learning framework. This approach addresses significant limitations in traditional analytical methods within environmental exposomics research. By integrating urinary biomarkers of heavy metals, PAHs, and VOCs into a novel EPEI, we demonstrated a significant correlation between cumulative pollutant exposure and hepatic lipid accumulation, particularly among metabolically vulnerable populations. The stacked ensemble model, which combines LASSO regression, SVM, neural networks, and XGBoost, outperformed conventional linear models in capturing nonlinear interactions and multicollinearity among pollutants, achieving interpretability through SHAP value analysis. Key findings identified 2-Hydroxynaphthalene as the most influential pollutant, with SHAP values exceeding 0.89 in meta-model analyses, underscoring its potential role in disrupting hepatic lipid homeostasis via oxidative stress and inflammatory pathways. Moreover, cobalt and specific VOCs metabolites, such as N-Acetyl-S-(2-carbamoylethyl)-L-cysteine, have been identified as significant contributors, highlighting the intricate nature of pollutant interactions in the pathogenesis of NAFLD.

The stratification analyses yielded three principal insights. First, the relationship between EPEI and PLF exhibited significant heterogeneity across various BMI categories, with the most pronounced effect observed in individuals with severe obesity [odds ratio (OR) = 2.66, 95% confidence interval (CI): 2.08–3.24]. This finding suggests an increased toxicity of pollutants in the context of adipose tissue dysfunction. Second, diabetes status modulated the exposure-risk relationship, with subgroups exhibiting IFG demonstrating greater diagnostic sensitivity compared to those with overt diabetes. This may reflect compensatory mechanisms active in the early stages of metabolic dysregulation. Third, hyperlipidemia significantly amplified the effects of pollutants (OR = 2.28 compared to 1.08 in non-hyperlipidemic individuals), aligning with experimental findings that associate lipid peroxidation with pollutant-induced hepatotoxicity. Demographic variables, notably age and alcohol consumption, exhibited paradoxical associations, with EPEI increasing with age but decreasing with alcohol intake. This suggests the presence of potential detoxification pathways or confounding lifestyle factors that warrant further investigation.

Methodologically, this study contributes to the field of environmental health research by establishing a framework for analyzing multi-pollutant effects using explainable machine learning techniques. The integration of SHAP values with ensemble modeling not only quantified the contributions of individual pollutants but also identified exposure thresholds for risk stratification, which is a critical step toward precision prevention. The superior performance of XGBoost in synthesizing base model predictions underscores the efficacy of gradient-boosted trees in managing high-dimensional exposure data. Nonetheless, certain limitations remain: the cross-sectional design limits causal inference, urinary biomarkers may not accurately reflect tissue-level pollutant burdens, and residual confounding from unmeasured covariates, such as dietary patterns, necessitates cautious interpretation.

These findings have significant public health implications. The development of EPEI offers a quantifiable metric for assessing environmental risk in NAFLD screening programs, especially for high-risk groups such as individuals with severe obesity and those experiencing early metabolic dysfunction. Future research should focus on the longitudinal validation of EPEI across diverse cohorts, mechanistic studies to elucidate the hepatotoxic pathways of 2-Hydroxynaphthalene, and intervention trials aimed at reducing pollutant exposure in metabolically compromised populations. By integrating exposomics with machine learning, this study establishes a foundation for multi-pollutant regulatory policies and personalized environmental health strategies in the context of the metabolic syndrome pandemic.

This study also has many limitations: single urine samples may not effectively capture chronic exposure due to variability in biomarker levels and quick elimination of some substances. This is especially true for studies on non-persistent chemicals like phthalates and bisphenols, where a single sample might only indicate recent exposure. To improve accuracy, repeated urine samples over time are recommended. Additionally, combining urine with blood samples can enhance exposure assessments, as blood samples provide insights into internal doses not fully captured by urine alone. This approach is beneficial for assessing VOCs, where the relationship between blood and urine levels can be complex (22, 23). The cross-sectional approach cannot preclude reverse causality, particularly given hepatic steatosis’ potential to modify xenobiotic metabolism. This critical limitation underscores the necessity for longitudinal designs incorporating serial pollutant and hepatic fat quantification to delineate exposure-disease causality.

## 5 Conclusion

This research develops a machine learning framework to evaluate the risks associated with multiple pollutants in non-alcoholic fatty liver disease (NAFLD), identifying 2-Hydroxynaphthalene as a significant hepatotoxicant and introducing EPEI as a quantifiable measure of exposure. The results underscore the metabolic vulnerabilities linked to obesity and early dysglycemia, thereby contributing to the development of precision prevention strategies.

## Data Availability

The original contributions presented in the study are included in the article/supplementary material, further inquiries can be directed to the corresponding author.

## Footnotes

^1^ https://wwwn.cdc.gov/Nchs/Nhanes/
