# Supplementary material for: The impact of multipollutant exposure on hepatic steatosis: a machine learning-based investigation into multipollutant synergistic effects
Source: Front Public Health. 2025 May 22;13:1598639. doi: 10.3389/fpubh.2025.1598639 (PMC12137238; doi:10.3389/fpubh.2025.1598639)

Supplementary Figure 1.Performance Discrepancy Between Training and Test Sets Across Models

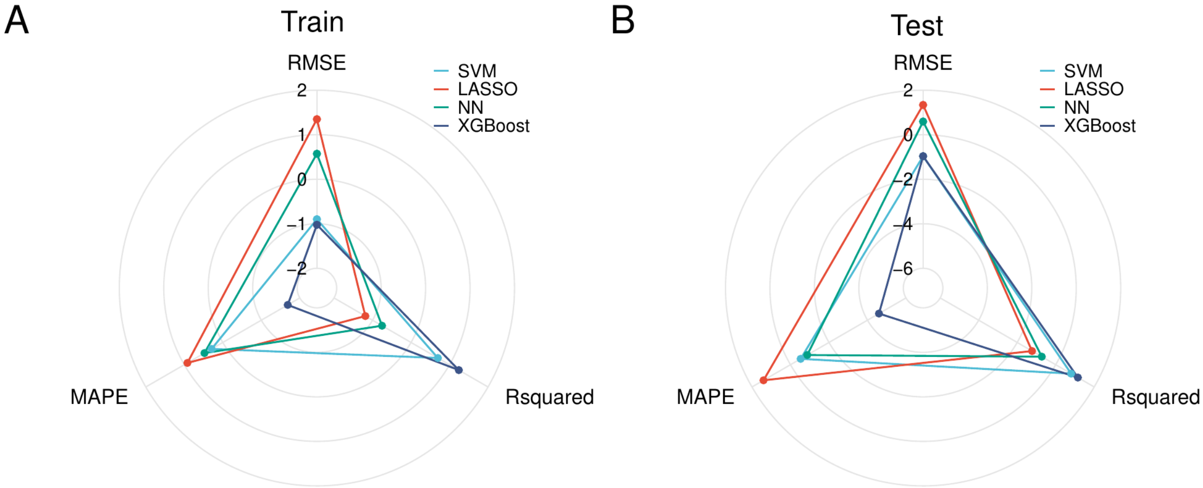

Supplement: Supplementary file 7 [file Image_1.pdf]
