# Supplementary figures and images for: The impact of multipollutant exposure on hepatic steatosis: a machine learning-based investigation into multipollutant synergistic effects
Source: Front Public Health. 2025 May 22;13:1598639. doi: 10.3389/fpubh.2025.1598639 (PMC12137238; doi:10.3389/fpubh.2025.1598639)

**Supplementary Figure 2. Feature Importance with Bootstrap Confidence Intervals**

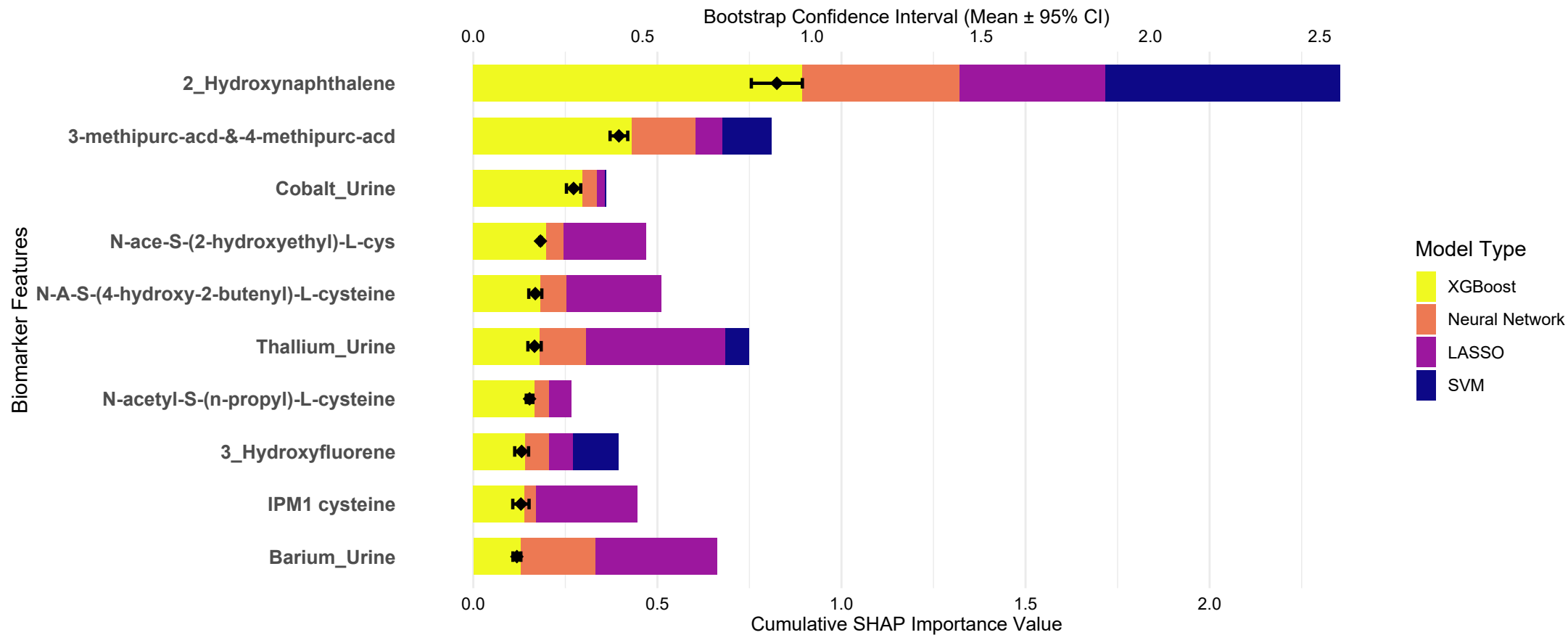

Supplement: Supplementary file 8 [file Image_2.pdf]

**Supplementary Figure 3.SHAP Ranking Correlation Across Models**

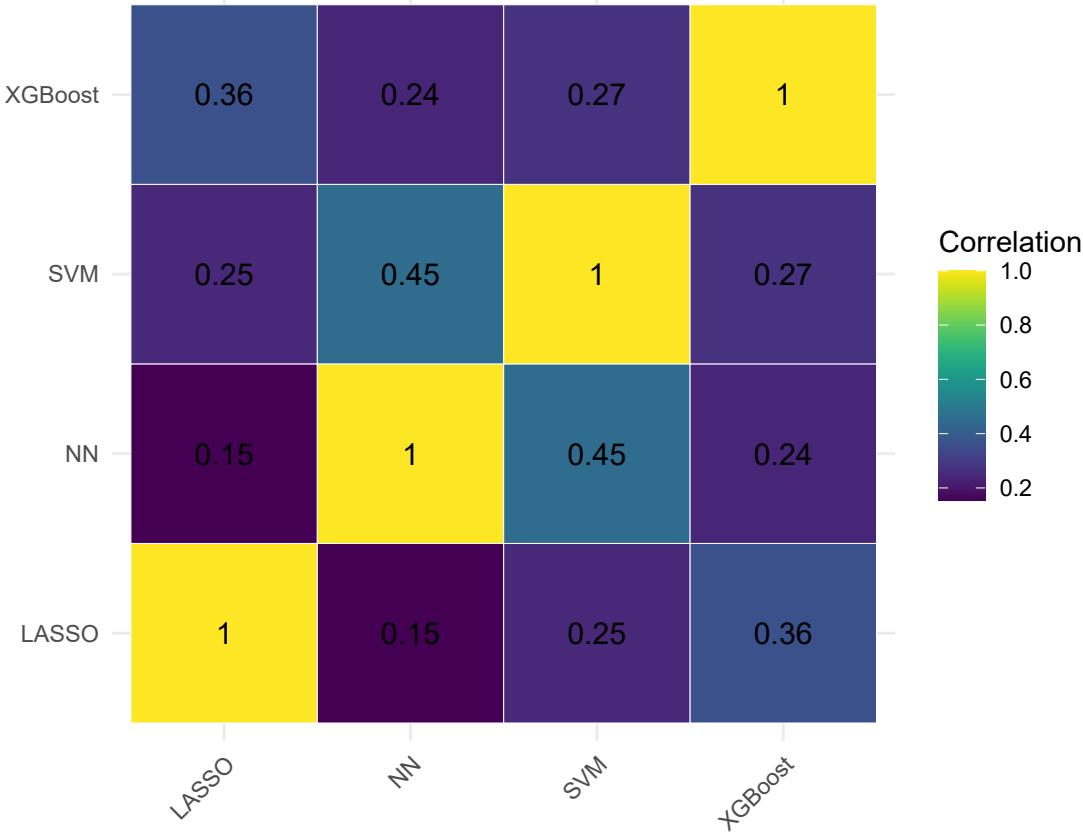

Supplement: Supplementary file 9 [file Image_3.pdf]
