# Supplementary material for: The impact of multipollutant exposure on hepatic steatosis: a machine learning-based investigation into multipollutant synergistic effects
Source: Front Public Health. 2025 May 22;13:1598639. doi: 10.3389/fpubh.2025.1598639 (PMC12137238; doi:10.3389/fpubh.2025.1598639)
Supplement: Supplementary file 10 [file Image_4.pdf]

Supplementary Figure 4.Linear Regression Analysis of EPEI and PLF.

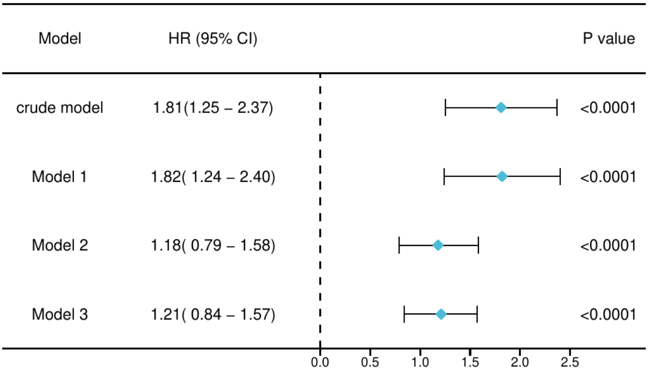

Crude model: no other covariates were adiusted
Model I: Adjust for age, education, poverty index
Model II: Adjust for age, education, poverty index, sex, hyperlipidemia, diabetes mellitus, body mass Index
Model III: Adjust for age, education, poverty index, sex, hyperlipidemia, diabetes mellitus, body mass Index, food intake(fruits, vegetables, grains, dairy, protein food, oils, solid fats , added sugar)
